# The HTLV-1 Virological Synapse

**DOI:** 10.3390/v2071427

**Published:** 2010-07-07

**Authors:** Mohamed Nejmeddine, Charles R.M. Bangham

**Affiliations:** Department of Immunology, Wright-Fleming Institute, Imperial College London, St. Mary’s campus, Norfolk Place W2 1PG, London, UK

**Keywords:** HTLV-1 virological synapse, viral proteins, cytoskeleton, polarization

## Abstract

Human T-lymphotropic virus-1 (HTLV-1) spreads efficiently between T-cells via a tight and highly organized cell-cell contact known as the virological synapse. It is now thought that many retroviruses and other viruses spread via a virological synapse, which may be defined as a virus-induced, specialized area of cell-to-cell contact that promotes the directed transmission of the virus between cells. We summarize here the mechanisms leading to the formation of the HTLV-1 virological synapse and the role played by HTLV-1 Tax protein. We propose a model of HTLV-1 transmission between T-cells based on the three-dimensional ultrastructure of the virological synapse. Finally, in the light of recent advances, we discuss the possible routes of HTLV-1 spread across the virological synapse.

## 1. Introduction

Human T-lymphotropic virus type 1 (HTLV-1) is a complex retrovirus, classified in the *Deltaretrovirus* genus of the subfamily *Orthoretrovirinae*. The majority (about 95%) of infected individuals develop no associated disease (asymptomatic carriers; ACs). However, in a small fraction of individuals, infection results in one of two types of disease: about 2–5% develop adult T cell leukemia/lymphoma (ATLL) [1], and a further 0.5–3% develop one or more of a range of inflammatory diseases. The most common of these conditions is HTLV-1-associated myelopathy/tropical spastic paraparesis (HAM/TSP), an inflammatory disease of the central nervous system [2,3].

Until recently, it was generally believed that HTLV-1 is latent *in vivo* [4–6]. However, there is significant evidence to the contrary [7,8]. The presence of high frequencies of activated HTLV-1-specific cytotoxic T lymphocytes (CTLs) [9,10] in the peripheral blood supports the hypothesis that the virus is not latent, *i.e.,* there is ongoing viral transcription, and that this is greater in patients with HAM/TSP than in ACs. Direct evidence for selective proliferation of HTLV-1-infected T cells was obtained by Asquith *et al*. (2007) by *in vivo* labeling with deuterated glucose [11].

In this review, we consider the mechanisms of cell-to-cell spread of HTLV-1. Following the discovery of the virological synapse (VS) in 2003, there have been significant advances in the understanding of the mechanism of formation of the synapse and in the locus of transfer of virions from cell to cell. We conclude that HTLV-1, as appears to be the case for HIV-1 and MLV, can be transferred from cell to cell both at sites of budding at the closely apposed plasma membranes at the VS and by lateral movement of preformed virions at, or near, the periphery of the cell-to-cell contact, where they may be protected in a ‘biofilm’ of extracellular matrix.

## 2. HTLV-1 cell tropism, cell-to-cell spread and the VS

HTLV-1 can infect a wide range of human cell types *in vitro* [12], but *in vivo* the virus is almost confined to the CD4^+^ T lymphocyte subset [13–16]. Furthermore, most of the malignancies induced by HTLV-1 are tumors of CD4^+^ T lymphocytes [17]. CD8^+^ T lymphocytes can also carry the virus, but at a consistently lower frequency than CD4^+^ T cells [18,19].

The conjunction of two observations led to the postulation of the VS. First, direct cell-to-cell contact is necessary for efficient transmission of HTLV-1 from an infected cell to a new host cell, both *in vitro* [20,21] and *in vivo* [22], where transmission depends on transfer of infected lymphocytes in breast milk [23–25], semen [26] or transfused blood products [27,28]. HTLV-1 virions are typically undetectable in the serum of infected individuals by RT-PCR. Virions are produced only by certain continuous *in vitro* T cell lines: fresh, naturally infected lymphocytes do not produce cell-free HTLV-1 particles. Furthermore, of the cell-free HTLV-1 virions that are produced by transfected T cells or continuous producer T cell lines, only one in 10^5^ to 10^6^ is infectious [29]. Second, HTLV-1-specific T cells are themselves infected more frequently with HTLV-1 than are T cells specific to other antigens. This preferential infection was evident in both CD8^+^ T cells [18] and CD4^+^ T cells [30]. These two observations raised the possibility that HTLV-1 transmission was assisted by the process of T cell antigen recognition. More precisely, HTLV-1 might spread across the ‘immunological synapse’ [31], the specialized area of contact that is formed between a lymphocyte and another cell in which distinct protein microdomains mediate adhesion, antigen recognition and secretion of cytokines or lytic granules. Confocal microscopy of conjugates formed spontaneously between *ex vivo* CD4^+^ cells from an HTLV-1-infected person and autologous (or allogeneic) lymphocytes revealed a structure at the cell–cell junction, which indeed resembled the immunological synapse [32]. Polarization of the adhesion molecule talin and the microtubule organizing center (MTOC) to the cell–cell junction was accompanied by accumulation of the HTLV-1 core protein Gag and the HTLV-1 genome at the cell-cell junction. After 2 h, both the Gag protein and the HTLV-1 genome were transferred from the infected to the uninfected cell [32].

A critical observation revealed the distinction between the immunological synapse and the structure formed between an HTLV-1-infected cell and another cell. In an immunological synapse, the MTOC in the responding T cell is polarized towards the antigen-presenting cell, such as a virus-infected cell. This polarization is triggered by engagement of the T-cell antigen receptor [33,34]. In contrast, in the cell-cell conjugates formed with an HTLV-1-infected cell, the MTOC was polarized inside the virus-infected cell, not towards it. The results are shown in Table 1 [32].

This observation showed that the mechanisms triggering the cytoskeletal polarization differed from the immunological synapse, and immediately suggested that the polarization was induced by HTLV-1 itself, perhaps in order to transmit viral material to the uninfected cell.

Inhibition of polarization and cell-to-cell transfer of HTLV-1 Gag protein at the cell-cell junction by the microtubule inhibitor nocodazole (30 μM) confirmed that the donor cell’s microtubule cytoskeleton plays a central part in cell-to-cell transmission of HTLV-1 [35,36]. Recently, Mazurov *et al.* [37] used an elegant new system of luciferase-expressing, replication-dependent vectors to quantify the infectivity of HTLV-1 and HIV-1 and the contribution of Tax protein to cell-to-cell transmission. These authors found that cell-to-cell transmission of HTLV-1 was >10^4^ times more efficient than cell-free virus, whereas cell-to-cell transmission of HIV-1 was only two-fold more efficient. Disruption of the microtubules with nocodazole reduced HTLV-1 co-culture infectivity by 85%, whereas HIV-1 infectivity was reduced by 60%. In addition, the authors showed that the induction of an immunological synapse between a Raji cell and a CD4^+^ target cell did not increase infection with either HIV-1 or HTLV-1 virus-like particles, suggesting that cell-to-cell infection requires the formation of a specialized VS [37]. These results confirm the exceptionally strong dependence of HTLV-1 transmission on cell-cell contact and the importance of cytoskeletal remodeling in the cell-to-cell transmission of both HTLV-1 and HIV-1.

Molecular triggers and intracellular pathways that cause the cytoskeleton polarization in HTLV-1-infected cells have now been identified (see below). Because both the mechanisms and the function of the HTLV-1-induced structure were distinct from the immunological synapse, the term ‘virological synapse’ was used [32].

## 3. Definition of the VS

A VS may be defined as a virus-induced, specialized area of cell-cell contact that promotes the directed transmission of the virus between cells. Many viruses are known to spread efficiently by cell-cell contact, but such spread typically takes place across normal, pre-existing cellular contacts. In contrast, a VS is actively induced by contact between an infected cell and another cell. The viruses that benefit most strongly from transmission by a VS are therefore those that infect mobile cells, such as leukocytes. The VS maximizes the efficiency of transmission and limits the exposure of the virus to host defense mechanisms, both in time and space.

## 4. Polarization of viral proteins at the VS

**Figure 1:**
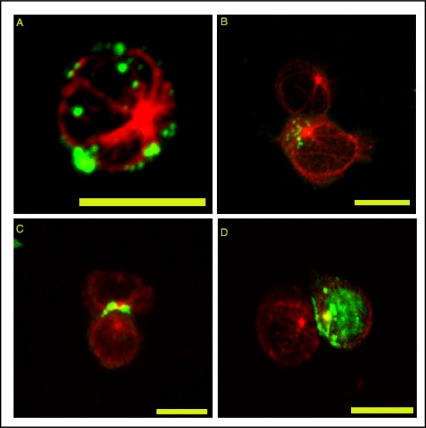
Polarization of HTLV-1 proteins and the microtubules network toward the VS formed between autologous CD4+ cells naturally infected with HTLV-1. **(A–C)** HTLV-1 Gag protein. **(D)** HTLV-1 Tax protein. **(A)** Isolated cell. **(C–D)** autologous CD4+ conjugates. Scale bars = 10μm. Originally published in Journal of Biological Chemistry [35].

**Figure 2:**
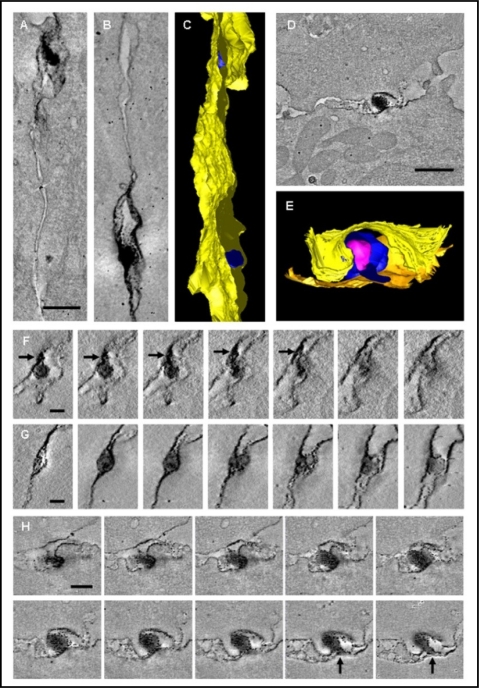
Three-dimensional ultrastructure of the VS. Enveloped HTLV-1 virions are trapped in multiple isolated synaptic clefts. Cell-to-cell transmission of HTLV-1 as observed in tomograms of the VS formed between HTLV-1 infected CD4+ T-cell (PBMC) and an autologous uninfected CD4+ T-cell as a target cell. These cells were stained against HTLV-1 Gag p19 matrix protein with a specific monoclonal antibody. **(A, B)** Projections along the z-axis of two subvolumes of the same tomogram showing viral transmission at two different locations. **(C)** Surface representation of the VS shown in (A, B): Several virions (blue) are trapped between the closely apposed plasma membranes (yellow). **(D)** Tomogram slice showing an HTLV-1 particle held between the cell membranes. **(E)** Surface representation of the virus transmission shown in D (cell membranes: yellow and orange, virus envelope: blue, virus core: magenta). **(F, G)** Tomogram slices through the two areas of virus transmission shown in (A) and (B), respectively, with a spacing of about 17 nm (F) and 25 nm (G) between subsequent slices. Black arrows indicate a protrusion linking the virus with the cell membrane. **(H)** Subsequent slices through the area of virus transmission shown in (D) with a spacing of about 17 nm. Black arrows indicate a protrusion linking the virus with the cell membrane. Scale bars: A, B 300 nm, D 500 nm, F, G 100 nm, H 200 nm. This figure was originally published in PLoS One [41].

The distribution of the HTLV-1 proteins Gag, Env and Tax can be visualized by confocal microcopy (Figure 1), and electron microscopy (Figure 2) in naturally infected cells [32,35,36,38–41]. In a single isolated cell (not making contact with a target cell) Gag is detected in the cytoplasm, forming large cytoplasmic inclusions whose precise nature remains unknown [42]. These inclusions appear to be randomly distributed around the periphery of the cell under the plasma membrane. Virions may also accumulate on the outside of the plasma membrane of an isolated cell, in the extracellular matrix ([93]: see Section 9 below). Env protein is uniformly distributed around the plasma membrane in an isolated cell; Tax protein is mostly nuclear but a significant fraction of Tax is present around the MTOC in association with the cis-Golgi compartment [35,43–45]. The distribution of Tax protein between the nucleus and the cytoplasm depends on the balance between ubiquitylation and sumoylation [44,46]. In cell conjugates formed between an HTLV-1 infected T-lymphocyte and autologous cells, the Gag and Env proteins are characteristically polarized toward the region of contact formed with the target cell [32,35,36,41]. In about 30% of cell-cell conjugates, a fraction of Tax also appears at the inner side of the plasma membrane in the region of cell-to-cell contact [35]: the function of Tax protein in this location is unknown. The mechanisms by which the HTLV-1 proteins are transported, sorted and delivered to the VS also remain unclear.

## 5. Polarization of the cytoskeleton to the VS

The formation of the VS is accompanied by the polarization of the MTOC in the infected cell toward the cell contact formed with the target cell (Figure 1). This polarization is a good indication of the establishment of a VS [32,35,36,47]. The polarization depends on the integrity of both actin and microtubule components of the cytoskeleton and requires the activity of the small GTPases Rac1 and Cdc42 [35].

As described above, the observation that the MTOC polarizes towards the cell-cell junction inside the HTLV-1-infected cell, rather than in a T cell responding to an antigen, revealed the distinction between the VS and the immunological synapse. The trigger that causes the microtubule polarization in the VS therefore cannot be delivered by the T-cell antigen receptor. It was thus postulated that two signals were required to trigger the MTOC polarization observed at the VS: one from cell-cell contact and one from the viral infection itself. Specifically, it was hypothesized that engagement of a receptor on the surface of the infected cell acted in synergy with a signal from an intracellular HTLV-1 protein to trigger the observed polarization.

### 5.1. Cell surface trigger of MTOC polarization at the VS

To identify the signal from cell-cell contact, latex beads coated with monoclonal antibodies were used to cross-link certain molecules on the surface of the infected T cell. As expected, cross-linking of the T cell receptor with an anti-CD3 monoclonal antibody caused efficient polarization of the MTOC towards the coated latex bead [47]. In fact, cross-linking of a number of other cell-surface molecules also triggers MTOC polarization [47], demonstrating the unusual mobility of the T cell’s microtubule cytoskeleton. However, engagement of either of two molecules, ICAM-1 (CD54) or the IL-2 receptor alpha chain (CD25), caused a significantly higher frequency of MTOC polarization in HTLV-1-infected cells than in uninfected cells. The importance of ICAM-1 in triggering MTOC polarization was corroborated by two further observations. First, a soluble cyclic peptide derived from the region of LFA-1 that normally binds to ICAM-1 selectively inhibited the observed preferential MTOC polarization in HTLV-1-infected cells towards an uninfected cell. Second, the selective MTOC polarization in HTLV-1-infected cells was abolished when the target cell lacked LFA-1, but was enhanced when the target cell expressed the constitutively actively form of LFA-1 [47]. The reason for selective polarization towards CD25 is less obvious: we have suggested that this results from the known physical association between CD25 and ICAM-1 in the T cell plasma membrane [48].

### 5.2. Intracellular trigger of MTOC polarization at the VS

The signal from ICAM-1 cross-linking was especially effective in triggering cytoskeletal polarization in cells infected with HTLV-1. What was the signal from HTLV-1 infection that acted in synergy with the ICAM-1 signal? The Tax protein of HTLV-1 was a strong candidate, because of its early and abundant expression during HTLV-1 replication and its known capacity to transactivate genes via the CREB, NF-κB and SRF pathways [44,46,49–53]. By transfecting a Tax expression plasmid into Jurkat T cells, we showed [35] that Tax is indeed sufficient to explain the observed preferential cytoskeletal polarization in HTLV-1-infected cells. We then used a series of mutants of the Tax protein to investigate the relationship between the signaling pathways activated by Tax, the intracellular localization of the Tax protein, and its ability to trigger MTOC polarization.

Mazurov *et al.* [37] have recently quantified the contribution of Tax to the efficiency of HTLV-1 transmission across the VS. Their results showed that Tax protein increased the efficiency of HTLV-1 transmission by more than 10-fold, confirming the earlier observations [35]. Remarkably, Tax also increased the efficiency of cell-to-cell transmission of HIV-1 by more than 10-fold, and the authors concluded that HTLV-1 Tax protein is a major determinant of the difference between HTLV-1 and HIV-1 transmission.

The cross-linking of ICAM-1 (reviewed in [54]) activates two distinct signaling pathways: one is RhoA-dependent [55], and the other involves Ras-MEK-ERK activation [56]. We previously showed that MTOC polarization in HTLV-1-infected cells is independent of RhoA GTPase activation [35]. Our recent data, obtained both in naturally infected lymphocyte and transfected Jurkat T-cells, confirms that MTOC polarization induced by the cross-linking of ICAM-1 depends on the Ras-MEK-ERK pathway [36]. This pathway is distinct from that activated by TCR engagement [36]. Indeed, HTLV-1 infection significantly reduced the frequency of MTOC polarization caused by cross-linking TCR (CD3) on the cell surface [47], which suggests a competition between the two pathways.

The identification of the major triggers involved in causing preferential microtubule polarization towards the VS provided a possible resolution of the long-standing paradox that HTLV-1 infection is almost confined to T cells *in vivo*, whereas it can infect almost any nucleated mammalian (and some avian) cells *in vitro*. First, the role of ICAM-1 can explain preferential transmission to LFA-1-positive cells, the great majority of which are T cells. Second, the role of microtubule polarization suggests that HTLV-1 has evolved specific mechanisms to subvert the unusual mobility of the T cell cytoskeleton in order to propagate. This in turn can explain the observation that, whereas HTLV-1 can infect other cell types *in vitro* such as epithelial cells and fibroblasts [29,57], it is not possible to propagate HTLV-1 in these cell types. That is, HTLV-1 can enter other cell types, but the infected cell is unable to pass the virus on to other cells.

In view of the observed sensitivity of the T cell’s microtubule cytoskeleton to engagement of a range of cell-surface molecules, it is likely that several ligand-receptor interactions contribute to cell-to-cell transmission of HTLV-1 [58,59]. However, the conclusion that ICAM-1 plays a particularly important role is reinforced by the observations that cross-linking of ICAM-1 can increase HTLV-1 protein expression [60], and conversely, HTLV-1 infection upregulates expression of ICAM-1 on the infected cell surface [61–64].

## 6. Transfer of viral protein and genome to target cells

Confocal microscopy was used to examine the distribution of HTLV-1 Gag and Env proteins and the HTLV-1 genome in fresh, unstimulated peripheral blood mononuclear cells (PBMCs) isolated directly from HTLV-1-infected individuals [32]. CD4^+^ or CD8^+^ T cells isolated from a HAM/TSP patient were allowed to form conjugates with T cells from a healthy uninfected donor for 120 min: in addition to accumulation of Gag p19 staining at the cell-cell junction, there was frequent Gag p19 staining in the cells derived from the uninfected donor [32,35,41]. In addition to Gag protein, HTLV-1 RNA was also transferred from infected cell to uninfected cell [32]. The HTLV-1 Gag p19 was observed to transfer from CD4^+^ T cells and CD8^+^ T cells to both CD4^+^ and CD8^+^ allogeneic T cells. This process may represent the initial establishment of HTLV-1 infection in a newly infected individual, which involves contact between allogeneic lymphocytes. Polarization of Gag complexes to the cell-cell junction and transfer to the uninfected cell were also observed in conjugates between CD4^+^ T cells and both B cells and NK cells [32]. The kinetics of HTLV-1 Gag transfer showed a peak intensity between 90 and 210 minutes after initiation of conjugate formation [36]. This kinetics closely resembles the kinetics of the cell-to-cell transfer of HIV-1-Gag recently reported [65]. HTLV-1 transfer is significantly reduced within this time frame either by depolymerization of the cytoskeleton (microtubules or microfilaments) or by inhibition of ERK phosphorylation and CREB activation in the infected donor T-cell [36].

## 7. Role of microtubule cytoskeleton in the cell-to-cell transfer of Gag protein

The microtubule organizing center (MTOC) is typically oriented to the area of cell-to-cell contact in lymphocyte conjugates where it lies immediately adjacent to the accumulation of HTLV-1 Gag protein [32,35,36]. This close apposition of polarized Gag molecules to the MTOC suggested that the microtubule cytoskeleton affected the polarization of Gag. In conjugates formed between infected and uninfected CD4^+^ T cells, depolymerization of the microtubule network blocked the polarization and transfer of Gag protein [32,35,36]. There is a significant association between MTOC polarization and Gag positivity in conjugates between autologous CD4^+^ and CD8^+^ T cells from an infected individual [32].

Recently, the HTLV-1 accessory protein p12 was reported to reduce the expression of ICAM-1/2, which in turn prevents the destruction of HTLV-1–infected cells by NK cells [66]. In addition p12 has been shown to induce LFA-1 clustering on the surface of infected T cells via a calcium-dependent signaling pathway, which is thought to promote HTLV-1 spread between T cells [67]. HTLV-1 Tax expression was also shown to strongly up-regulate ICAM-1 expression [61]. Thus, HTLV-1 appears to regulate the expression of adhesion molecules at the surface of infected T-cell using both Tax and p12 protein. However, the early expression of Tax protein and its powerful effect on ICAM-1 suggests that Tax will dominate – and so upregulate ICAM-1 – in the early stages of the infectious cycle, so promoting the formation of the VS.

The association of Tax protein with the MTOC region in the infected T cell and its ability to activate CREB pathway are both required for the polarization observed during the formation of the HTLV-1 VS [36]. Tax also binds directly to the protein kinase MEKK1 [68], and this interaction might play a part in the synergistic induction of MTOC polarization by Tax and ICAM-1. However, Tax protein interacts with a large range of cellular proteins, including Ras p21 proactive 2 and cdc42/Rac effector kinase [69], which are a part of the Ras activation pathway.

## 8. Ultrastructure of the VS: Electron tomography

We investigated the three dimensional ultrastructure of the HTLV-1 VS using electron tomography (Figure 2) of immunostained conjugates formed between CD4^+^ T cells from HTLV-1-infected individuals and conjugates between MS9 cells (a chronically HTLV-1-infected cell line) and Jurkat cells [41].

Enveloped HTLV-1 particles were identified in an intercellular space or “synaptic cleft” in the VS formed between a naturally infected lymphocyte and an autologous target lymphocyte (Figure 2). Each synaptic cleft is characteristically bounded by the tightly apposed membranes of the infected and target cells, with a membrane-membrane distance of ∼20 nm (median 25.7 nm). This distance is consistent with measurements of intercellular separation between lymphocytes and target cells, and with data on the conformation of ICAM-1/LFA-1 binding from electron microscopy and X-ray crystallography [70–72]. However, the ultrastructural studies of the VS show that in the case of HIV-1 the intercellular space is less tight than in the HTLV-1 VS and is therefore likely to be accessible to inhibitors both during and after virus assembly [73].

The HTLV-1 VS typically contains more than one synaptic cleft, spatially distinct and separated by areas of close membrane–membrane apposition (Figure 2, A–C). The membrane of the synaptic pockets presents sites of virus budding, and the pockets contain enveloped HTLV-1 particles [41]. This contrasts with the immunological synapse where secretory lysosomes are secreted into a single synaptic pocket in the cytotoxic synapse [74,75]. The size of HTLV-1 particles that originate from naturally infected CD4^+^ T cells has a wide range (62 nm–173 nm), with a peak at about 100 nm similar to the variability in size of HIV-1 particles [76,77]. The directional nature of the process is indicated by the polarization of Gag and Tax proteins, and the MTOC [32,35,47]. The accumulation of mitochondria near the VS (Figure 2, D) again resembles the immunological synapse [41,75].

**Figure 3:**
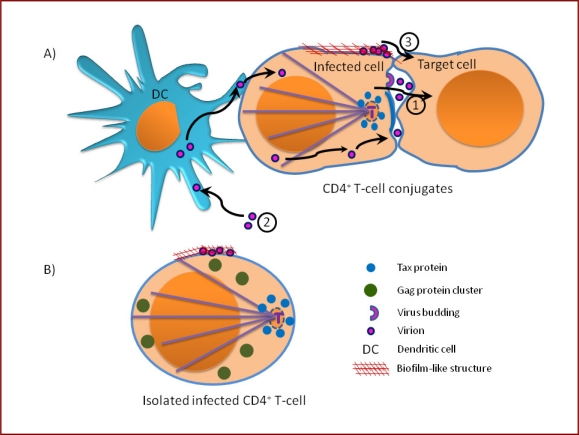
Illustration of possible routes of HTLV-1 spread between cells *in vivo.* **(A)** cell-to-cell transmission via intercellular synaptic cleft surrounded by a tight cell-cell contact between the donor and recipient cell, **1**. Cell-free particles can be internalized by dendritic cells and are then transferred to lymphocyte only by cell-to-cell contact, **2**. Virus particles can be retained on the cell surface in a biofilm-like-structure before lateral transfer to the recipient outside the cell-cell contact region, **3. (B)** In an isolated HTLV-1-infected lymphocyte, the viral proteins are not polarized.

The ultrastructure of the HTLV-1 VS reconciles the requirement for cell contact and HTLV-1 Env protein for the spread of the virus, with the lack of detection of cell-free virions in the serum. Whereas most viruses spread by releasing large numbers of virions from the infected cell, HTLV-1 uses the mobility of the host cell to propagate from cell to cell. Sequestration in the synaptic cleft presumably allows efficient transfer of small numbers of virions, and may give the virions a degree of protection from components of the immune response (complement system and antibodies) (Figure 3, A1).

## 9. Recent advances

New routes have been recently described that may contribute to HTLV-1 spread *in vivo*. Jones and co-authors investigated the role of human dendritic cells (DCs) in the transmission of HTLV-1 [78]. DCs are potent antigen-presenting cells that play a central role in initiating the immune response. However, infection of DCs can impair their ability to mount an appropriate immune response. Indeed, many viruses infect DCs to facilitate their transmission including the retrovirus HIV-1 and the mouse mammary tumor virus [79–81]. Some viruses use the trafficking proprieties of DCs to facilitate their transport from the periphery to lymph nodes where they infect target cells.

Jones *et al*. have shown that myeloid DCs (mDC) and plasmacytoid DCs (pDC) are efficiently and reproducibly infected *in vitro* with cell-free HTLV-1 virions released by chronically infected T cell lines (MT2, DB1) [78]. The infected DCs can rapidly transfer HTLV-1 to autologous primary CD4^+^ T-cells, resulting in a chronic productive infection of CD4^+^ T cells *ex vivo.* The DC-T cell transmission of HTLV-1 is reduced by blocking Neuropilin-1 (NP-1) and heparin sulfate proteoglycans (HSPGs) [78], two molecules involved in the initial interaction of HTLV-1 with CD4^+^ T-cells [82–85] and in the DC-T cell interaction [86,87]. DC-specific ICAM-3-grabbing nonintegrin (DC-SIGN) was also shown to facilitate the interaction of dendritic cells with HTLV-1-infected cells [88]. Recently it was reported that blocking any one of four molecules HSPGs, NRP-1, GLUT-1 or DC-SIGN led to reduction of virus binding to the cell; however, HTLV-1 transmission from DCs to T cells was mediated primarily by DC-SIGN [89]. Based on their findings and on the studies reporting the presence of viral proteins [90] and genome [91,92] in DCs isolated from HTLV-1-infected individuals, Jones *et al*. suggested that cell-free virus can also infect DCs *in vivo* (Figure 3, A2). It is possible that DCs play an important part in the early stages of HTLV-1 infection of a new host, by efficiently acquiring the virus and disseminating it to T cells. This system provides a new approach for dissection of the early events in HTLV-1-induced transformation and HTLV-1 infection.

Pais-Correia *et al*. have reported evidence that HTLV-1 can also be transferred from cell to cell at the VS via biofilm-like extracellular viral assemblies [93]. By using electron and light microscopy analysis they detected extracellular viral clusters at the cell surface of HTLV-1 infected cell after overnight incubation *in vitro*. These viral assemblies are carbohydrate-rich structures, suggesting the involvement of matrix-linker proteins in the adhesion and attachment of HTLV-1 to the surface of infected cells. The authors concluded that the extracellular matrix component and cellular lectins together generate a cocoon-type structure that concentrates virions in a confined protective environment to escape the immune response. Consistent with the tomographic studies of HTLV-1-VS [41] the authors observed that infected T cells formed a tight contact with the target cell. However, their microscopic analysis suggested that many virions cluster overlapped the cell contact, bridging the gap between the two T cell surfaces [93]. Extensive washing or heparin treatment of chronically infected cells and primary infected CD4^+^ cells reduces their capacity to infect reporter target cells [93]. In contrast with an earlier report [78], the cell-free HTLV-1 particles recovered from the cell surface clusters by extensive washing and heparin treatment were able to infect target T cells, although less efficiently than by cell-to-cell contact at the VS.

Recent studies on Murine Leukemia Virus (MLV), a retrovirus related to HTLV-1, have quantified the sequential assembly and transmission events for individual viral particles in living cells [94,95]. The authors showed that MLV Gag protein is present in 10-fold greater amounts at the cell-cell contact area compared to the periphery and that the *de novo* assembly of virions is highly polarized toward the zone of cell-to-cell contact [94]. They also observed that a fraction of 30% of MLV particles is retained at the infected cell surface after completion of assembly, and they named this fraction “surfacing viruses”. These virions are competent for transmission to infected target cells after the establishment of a physical cell-to-cell contact [95]. This fraction may represent a reservoir of infectious virus particles that can be sequestered and later passed on to uninfected target cells upon the establishment of cell-to-cell contacts.

We conclude that HTLV-1, like other retroviruses (notably HIV-1 and MLV), can be transferred between cells at more than one locus at the VS. The relative magnitude of cell-to-cell transmission of virions that bud within the cell contact zone and preformed virions at the periphery of the cell contact zone remains to be quantified (Figure 3, A3); this ratio may depend on the time interval between the onset of viral antigen expression and the formation of a cell-cell contact.

To identify and quantify the mechanisms involved in the formation and function of the HTLV-1 VS, experimental work *in vitro* has focused chiefly on two-cell conjugates. However, it is likely that, *in vivo*, an HTLV-1-expressing T-cell will make contact with more than one cell simultaneously in the low-velocity, cell-rich environment of the lymphoid circulation. HIV-1 has indeed been shown to spread in culture from one infected cell to more than one recipient cell: the authors called the multiple contact a polysynapse [96]. These rosette-like structures, displaying Gag accumulation at each cell contact, were observed in infected lymphocytes and DCs. Microscopic analysis suggests that the cell contacts in the polysynapse can be formed simultaneously rather that one after another. Multifocal capping of assembled budding virions required for polysynapse formation is promoted by tetraspanins and plasma membrane mobility via lipid rafts. The actin and microtubules are also involved because nocodazole, cytochalasin D and latrunculin B inhibit the formation of polysynapses. The description of the virological polysynapse recalled the studies of multiple immunological synapse formation by T cells that interact simultaneously with cells presenting different antigenic stimuli [97]. These studies showed that the MTOC moves repeatedly between the different cell-cell contacts; eventually the secretory machinery becomes selectively polarized toward the antigen-presenting cell that provides the strongest stimulus. The intensity of molecular translocation at the different synapses reflects the strength of signals received via the T-cell receptor and accessory molecules [97].

The present studies carried out *in vitro* and *ex vivo* indicate that HTLV-1 may use more than one route to spread between CD4^+^ T-cells. However, it is clear that efficient propagation of HTLV-1 depends on the close contact whose formation is triggered by the virus infection in the VS. While it is difficult to completely exclude the possibility of cell-free virion transmission of HTLV-1 *in vivo,* cell-free HTLV-1 particles are typically undetectable in serum even by RT-PCR, and transfusion of plasma or the cell-free blood product does not transfer HTLV-1 infection. It is possible that transfer of viral particles at the periphery of the VS involves the same molecular mechanisms that cause the polarization of HTLV-1 protein and the cytoskeleton, as described above. However, this remains to be tested.

## 10. Mechanism of HTLV-1 entry: Comparison of two possible mechanisms of HIV transmission

The mechanism by which HTLV-1 penetrates the target cells is yet to be established. Endocytosis is an obligatory entry step for enveloped viruses whose fusion proteins are activated by acidic pH [98]. However, HIV can mediate fusion between adjacent target cells (“fusion from without”) and HIV Env expressed on effector cells promotes fusion with target cells at neutral pH [99].

### 10.1. Fusion from without at the cell membrane in intercellular pockets at the VS

Certain enveloped viruses such as herpes simplex virus 1 (HSV-1), Sendai virus, and many retroviruses, including HIV, have pH-independent fusion proteins and can therefore penetrate into cells by fusing directly with the plasma membrane. It is generally assumed that fusion events at the plasma membrane lead to productive infection, although this is difficult to prove because virus particles are also continuously endocytosed [98].

Penetration of enveloped viruses occurs by membrane fusion catalyzed by fusion proteins in the viral envelope. The machinery involved is rather simple, at least when compared to the apparatus needed for intracellular membrane-fusion events. One reason for simplicity is that viral fusion factors are used only once. Fusion activity is triggered by cues in the form of receptor binding or low pH. They induce, as a rule, irreversible conformational changes. Membrane fusion is an elegant and effective way to deliver viral capsids into the cytosol. No macromolecular assemblies need to pass through a hydrophobic membrane barrier. The underlying principle is the same as in intracellular membrane traffic; the viral envelope is a “transport vesicle”, and the capsid is the cargo [100].

### 10.2. Endocytosis

The endocytic entry route gives many advantages to the virus: (i) the endocytic vesicles are designed to traverse the barriers imposed by the cortical cytoskeleton and the highly structured cytoplasm, so endocytosis gives the virus efficient access into the cytoplasm; (ii) the incoming viruses are exposed to compartmental environments that differ from the extracellular milieu, in particular the mildly acidic pH in endosomes provides an essential signal that triggers penetration and uncoating; (iii) if the penetration is lytic, endosomal membrane lysis is likely to be less damaging to the cell than lysis at the plasma membrane. However, it is possible that endocytosis leads to delivery of the virus to the lysosome, a degradative compartment and a dead-end for most viruses. This is why viruses have adapted to carefully adjust the threshold pH for activation to match that of early (pH 6 to 6.5) or late endosomes (pH 5 to 6) [100]. The early and late endosomes constitute distinct entry sites: this has been confirmed with dominant negative mutants of endosome-associated small guanosine triphosphatases (GTPases) [101]. A constitutively inactive mutant of Rab5 (early endosome) blocked the entry of both Semliki Forest virus (pH 6.2) and influenza virus (pH 5.4), whereas the corresponding Rab7 mutant (late endosomes) only blocked influenza virus entry. The progress of individual virus particles through endocytic compartments can be tracked with real-time video microscopy [65,102]. Individual fluorescent virus particles can be observed to bind to the cell surface, diffuse along the membrane, get trapped in coated pits or caveolae, enter by endocytosis, and move along microtubules. With the use of specific fluorescent dyes, the acidification of virus particles and the fusion of the viral envelope with cellular membranes can also be monitored.

## 11. Summary and Conclusions

1. A VS may be defined as a virus-induced, specialized area of cell-cell contact that promotes the directed transmission of the virus between cells.
2. HTLV-1 relies almost exclusively on cell-to-cell transmission to spread, both within the host and between hosts. Only dendritic cells can be efficiently infected with cell-free HTLV-1: this route may be important during initial acquisition of infection. However, DCs can infect T cells only by cell contact.
3. The HTLV-1 VS has a large area of close (about 26 nm) apposition of the plasma membranes of the infected cell and the target cell; there is no evidence of fusion between the plasma membranes of the two cells. The viral proteins Gag, Env and Tax are polarized at the cell-cell junction.
4. The integrity and function of both actin and microtubule cytoskeletons are required for the formation of the HTLV-1 VS and for transfer of HTLV-1 between cells. The MTOC is polarized to the cell-cell junction.
5. HTLV-1 Tax protein acts in synergy with crosslinking of ICAM-1 on the infected cell surface, to cause polarization of the infected cell’s microtubule cytoskeleton to the VS. This polarization requires the presence, in the vicinity of the MTOC, of a Tax molecule competent to activate the CREB pathway. The ERK pathway is also required for MTOC polarization at the VS.
6. An HTLV-1-infected cell can form more than one VS simultaneously (a “polysynapse”). The multiple contacts strongly resemble the multiple immunological synapses previously demonstrated. As in the multiple immunological synapses, it is likely that the dynamic microtubule cytoskeleton of the T cell moves between the respective cell contacts in a multiple VS.
7. The closely apposed cell membranes at the VS are interrupted by multiple intercellular clefts or ‘pockets’. Immunoelectron tomography has shown HTLV-1 Gag staining localized under the plasma membrane in these pockets. The pockets contain Gag-staining particles within the intercellular clefts consistent with the size and morphology of virions. Virions may be transferred to the target cell either across these clefts, or at the periphery of the cell contact, or (probably) both.
8. Env protein is required for infectivity of HTLV-1. Both endocytosis and fusion from without (virion fusion with the plasma membrane) may contribute to infection of the target cell; the relative importance of the two routes remains unknown.
9. The HTLV-1 VS appears to differ from the HIV-1 VS in the closeness of the cell-to-cell contact and the enhancing role of HTLV-1 Tax protein.

## Figures and Tables

**Table 1:** HTLV-1-infected cells polarize their MTOCs to the cell-cell junction in CD4+ T-cell conjugates. Two experiments were performed, each with fresh *ex vivo* CD4+ T cells from an unrelated HTLV-1-infected subject. Conjugates were allowed to form for 30 min (Subject 1) or 60 min (Subject 2), then were fixed and stained for HTLV-1 Gag p19 and tubulin alpha. Only conjugates containing two cells were counted. The figures denote the number (percentage) of cells whose MTOC was polarized to the cell-to-cell junction. Odds ratio^(a)^ of MTOC polarization in Gag p19+ cells, comparing the number of polarized MTOCs with (polarized + not seen) MTOCs. This table was originally published in the material accompanying reference [32].

MTOC orientation	Uninfected control subject	HTLV-1-infected subjects			
		Subject 1		Subject 2	
		Gag p19^−^	Gag p19^+^	Gag p19^−^	Gag p19^+^
Polarized %	79 (18.7)	85 (25.9)	**163 (58.2)**	45 (22.0)	**59 (53.2)**
Not polarized %	322 (76.3)	**217 (66.2)**	111 (39.6)	**160 (78.0)**	52 (46.8)
Not seen	21 (5)	26 (7.9)	6 (2.1)	0 (0.0)	0 (0.0)
Total	422 (100.0)	328 (100.0)	280 (99.9)	205 (100.0)	111 (100.0)
Odd ratio ^a^ (95% confidence interval) χ^2^ =(P<<0.001)	–	3.98 (2.83 – 5.61) χ^2^ = 63.9 (P<<0.001)		4.02 (2.45–6.64) χ^2^ = 30.4 (P<<0.001)	
		Subject 1 and subject 2 combined: **4.07** (3.07 – 5.39) χ^2^ = 99.0 (P<<0.001)			
